# Artificial Intelligence—A Good Assistant to Multi-Modality Imaging in Managing Acute Coronary Syndrome

**DOI:** 10.3389/fcvm.2021.782971

**Published:** 2022-02-16

**Authors:** Ming-hao Liu, Chen Zhao, Shengfang Wang, Haibo Jia, Bo Yu

**Affiliations:** ^1^Department of Cardiology, The 2nd Affiliated Hospital of Harbin Medical University, Harbin, China; ^2^The Key Laboratory of Myocardial Ischemia, Chinese Ministry of Education, Harbin, China

**Keywords:** acute coronary syndrome, artificial intelligence, machine learning, computed tomography, magnetic resonance, coronary angiography, intravascular ultrasound, optical coherence tomography

## Abstract

Acute coronary syndrome is the leading cause of cardiac death and has a significant impact on patient prognosis. Early identification and proper management are key to ensuring better outcomes and have improved significantly with the development of various cardiovascular imaging modalities. Recently, the use of artificial intelligence as a method of enhancing the capability of cardiovascular imaging has grown. AI can inform the decision-making process, as it enables existing modalities to perform more efficiently and make more accurate diagnoses. This review demonstrates recent applications of AI in cardiovascular imaging to facilitate better patient care.

## Introduction

Acute coronary syndrome (ACS) is a common type of coronary artery disease, which often leads to devastating consequences (1–3). Therefore, researchers and clinical practitioners have devoted countless efforts to the prevention, diagnosis, treatment, and rehabilitation of it. Various imaging modalities have emerged in this context, including non-invasive methods such as coronary computed tomographic angiography (CCTA), cardiac magnetic resonance (CMR), single-photon emission computed tomography (SPECT) myocardial perfusion imaging, and invasive approaches such as coronary angiography (CAG), intravascular ultrasound (IVUS), optical coherence tomography (OCT), fractional flow reserve (FFR), and near-infrared spectroscopy (NIRS), etc. Even though patients with ACS benefit comprehensively from the application of the above-mentioned imaging modalities, there are still gaps in understanding.

**Figure 1:**
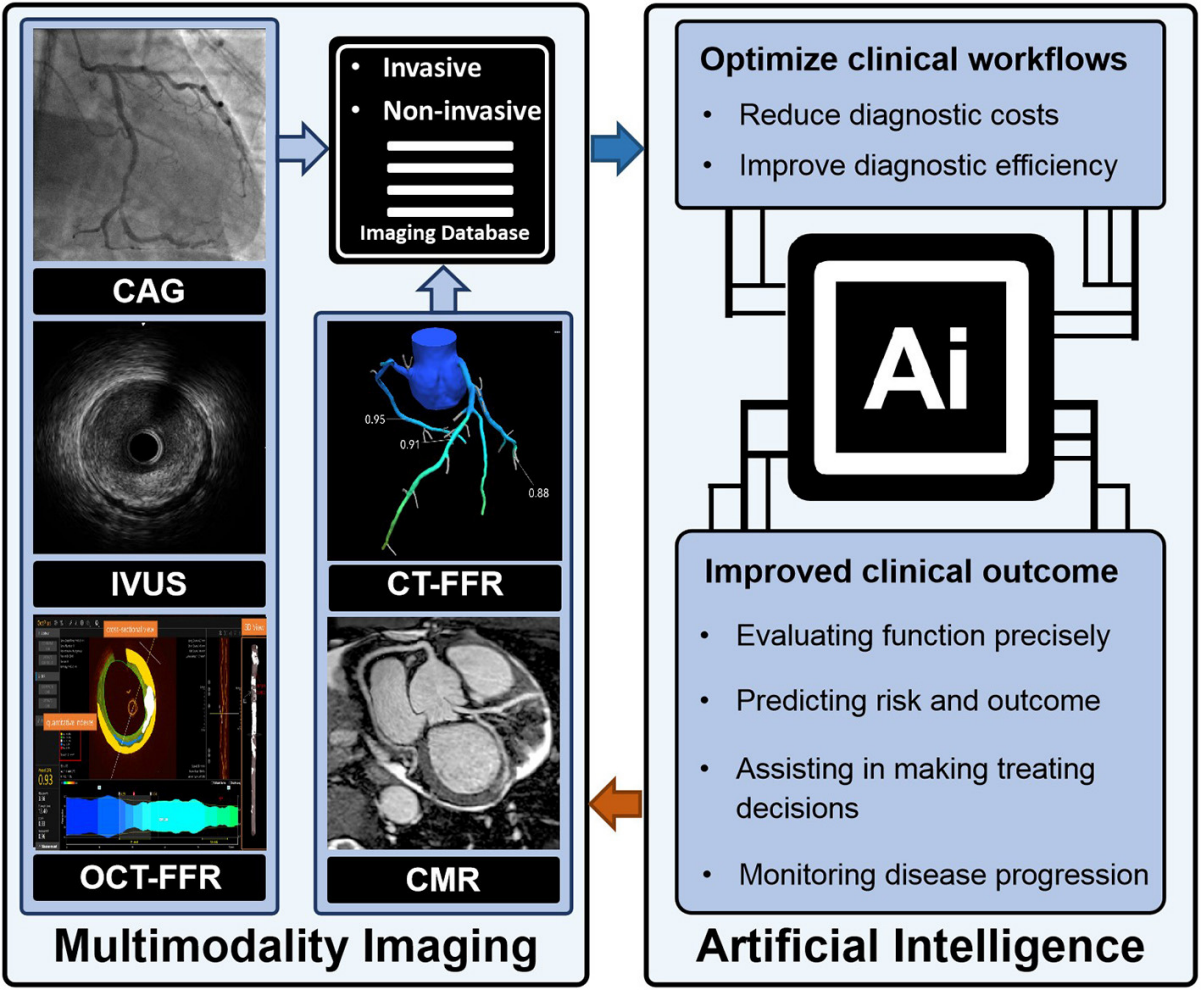
AI and imaging.

Artificial intelligence (AI) is a computerized program that resembles the human brain by collecting and processing data (4). With proper training, a variety of tasks that used to be undertaken by people can now be finished by AI. An overview of commonly used machine learning algorithms is shown in Table 1. Everyday life has already been extensively “infiltrated” by AI. A tremendous amount of work has already been put into cardiovascular imaging that combines AI, hoping to help clinicians achieve better healthcare for patients with ACS in particular. To date, scientific researchers have successfully developed AI to process imaging data, support diagnosis, interpret an image, provide treatment advice, recognize patterns of disease, and so on (Figure 1). Applications of AI in non-invasive modalities are summarized in Table 2, while applications in invasive ones are in Table 3. This review discusses recent applications of AI in cardiovascular imaging modalities related to ACS.

**Table 1:** Overview of common AI algorithms.

**Algorithm**	**Description**	**Illustration**
Convolutional neural network (CNN)	A typical CNN consists of convolutional layer, max pooling layer and fully connected layer. Convolutional layer extracts features in the image, max pooling layer downsamples the features. Usually the former two layers repeat many times. Fully connected layer classifies the features from the former 2 layers	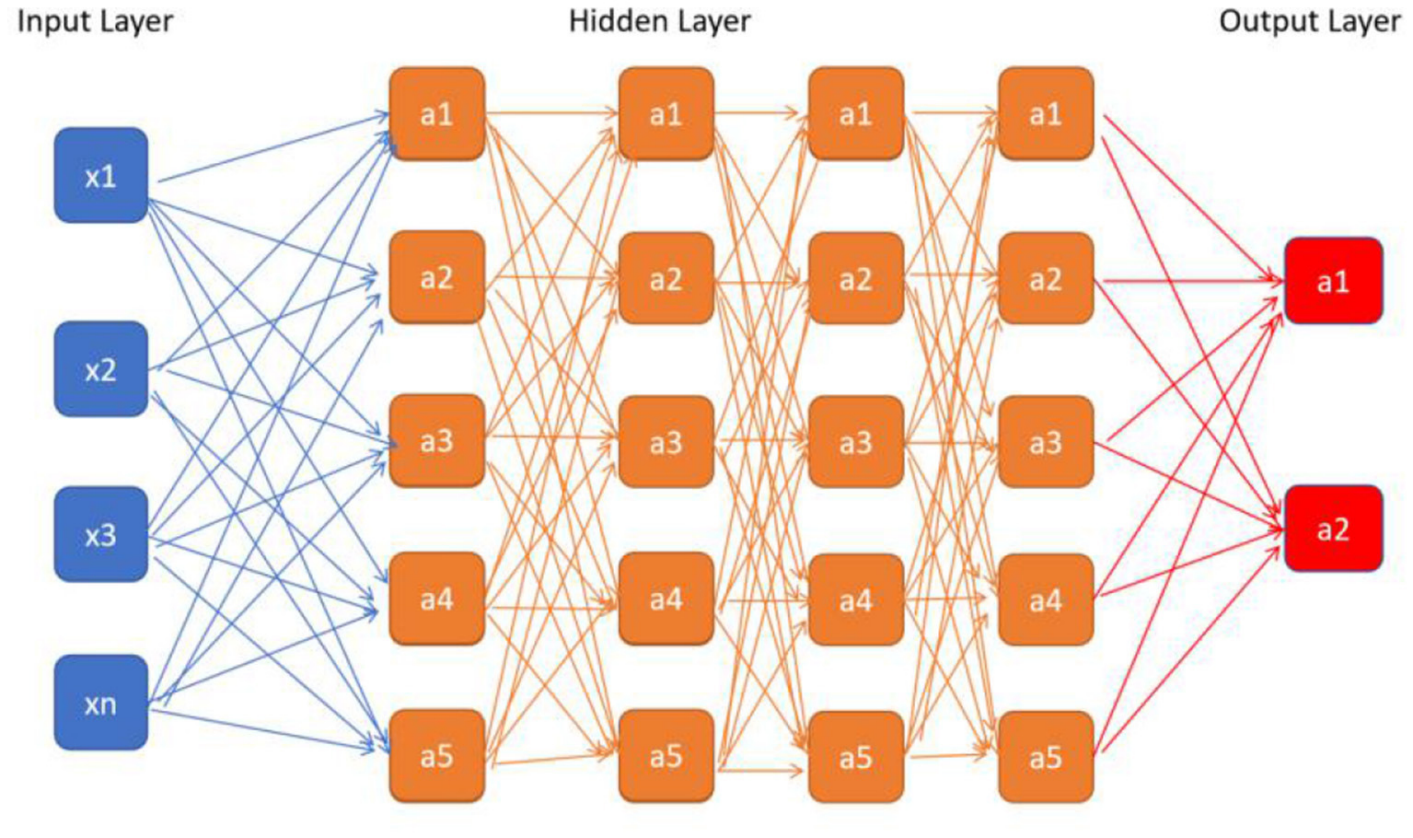
eXtreme Gradient Boosting (XGBoost)	Based on gradient boosting decision tree, highly effective and flexible. It is a sparsity aware algorithm and a weighted quantile sketch for approximate learning	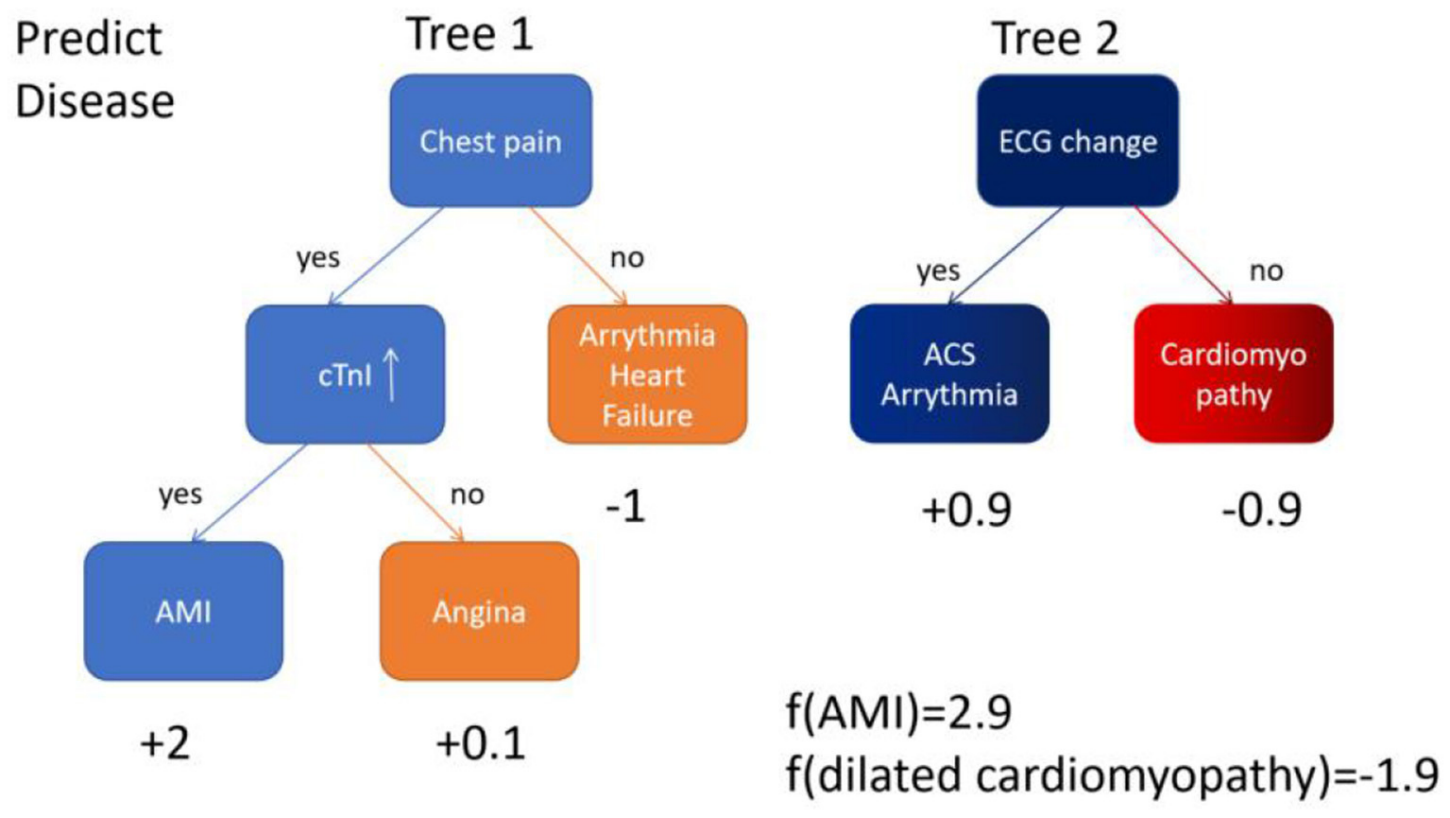
Random forest (RF)	A supervised machine learning classifier. Consisted of many decision trees, it induces random feature selection during the training process. It output a single result after combining multiple decision trees	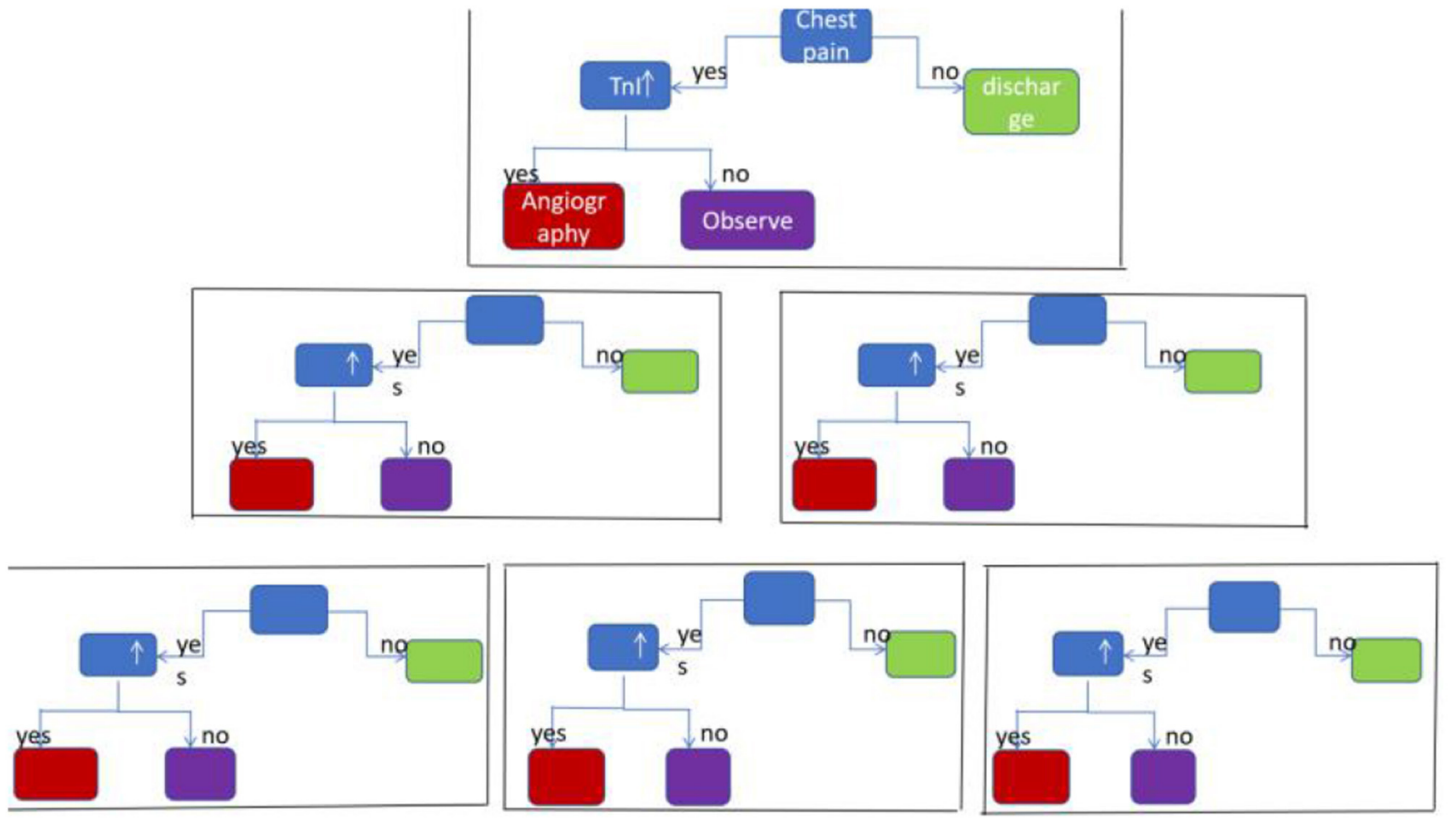
Support vector machine (SVM)	A supervised machine learning method designed to solve two-group classification problem. It aims to find a hyperplane to mostly separate data of two groups	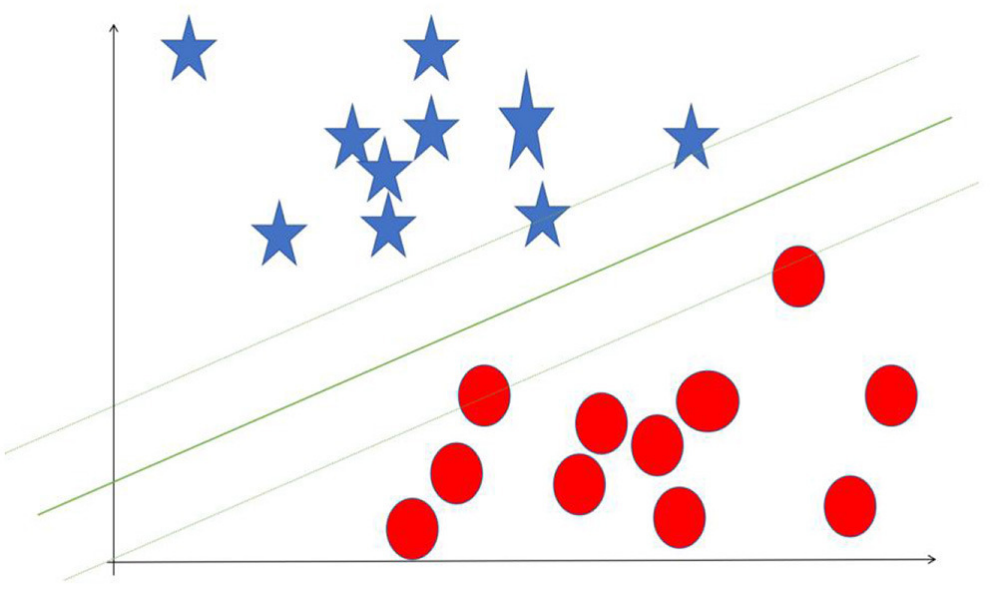

**Table 2:** Applications of AI in non-invasive modalities.

**References**	**Modality**	**Purpose**	**Samples**	**Algorithm**	**Results**
Liu et al. (5)	CT	Investigate CT-FFR as an alternative in deciding on intervention	243 patients	Tree-structured RNN	MACE rate with a CT-FFR value ≤ 0.8 (2.9%) similar to that of CAG-guided interventions (3.3%) (*p* = 0.838)
Duguay et al. (6)	CT	Investigate the prognostic value of CT-FFR	48 patients	Deep neural network	CT-FFR ≤ 0.80 has a HR of 1.56 [1.01–2.83], (*p* = 0.048) to predict MACE
Eberhard et al. (7)	CT	Evaluate feasibility and clinical role of CT-FFR	56 patients	Deep neural network	Agreement of 81% in CT-FFR and clinical diagnosis of ACS
Zeleznik et al. (8)	CT	Validate an automatic method of quantifying coronary calcium	20,084 patients	Deep CNN(fully) of U-net architecture	Spearman's correlation of 0.92 (*P* < 0.0001) to manual measurement.Strong predictor of cardiovascular events (multivariable-adjusted HR up to 4.3)
Qiao et al. (9)	CT	Investigate if FSS_CTA_ can predict outcome in three vessel CAD patients	227 patients	Deep neural network	FSS_CTA_ (OR = 1.21, *P* = 0.001). Predictive accuracy for MACE of FSS_CTA_ AUC: 0.81, *P* = 0.01
Al'Aref et al. (10)	CT	Identify culprit lesion precursors among ACS patients based on CT-based plaque characteristics	468 patients	XGBoost	ML model's AUC of identifying culprit lesion precursors of 0.774(CI: 0.758–0.790)
Lin et al. (11)	CT	Determine whether CT-based PCAT can distinguish patients with AMI with those with stable angina or no CAD	180 patients	XGBoost	AUC 0.87 in discriminating AMI
Tamarappoo et al. (12)	CT	Assess an ML risk score to predict long-term hard cardiac events	1,069 patients	XGBoost	The ML risk score AUC 0.81
Yang et al. (13)	CT	Lumen narrowing and plaque characteristics to predict ischaemia and outcome	1,013 vessels	Boruta and hierarchical clustering	Six features predicting low FFR AUC 0.797 (*P* < 0.001). AUC of them predicting vessel-oriented composite outcome 0.706 (*p* = 0.031)
Oikonomou et al. (14)	CT	Find FRP that can predict MACE	1,777 patients	RF	The coronary FRP signature can predict MACE (C-statistic 0.77 [95% CI: 0.62–0.93])
Larroza et al. (15)	CMR	Texture features to differentiate AMI	44 patients	RF SVM	Polynomial SVM AUC of 0.86 ± 0.06 on LGE MRI, AUC of 0.82 ± 0.06 on cine MRI
Schuster et al. (16)	CMR	Investigate feasibility and prognostic implications of AI-based software analysis	1,017 patients	suiteHEART, v4.0.6; Neosoft	Manual and automated volumetric assessments'impact on outcome (manual: HR, 0.93; automated: HR, 0.94)
Ma et al. (17)	CMR	Feature study on CMR to diagnose myocardial injury in AMI	68 patients	ML	Radiomics and T1 values AUC of 0.88 (training set) and 0.86 (test set)in diagnosing MVO
Knott et al. (18)	CMR	Explore the prognostic significance of MBF and MPR	1,049 patients	CNN	MBF: adjusted HR for death and MACE 1.93 and 2.14 MPR: adjusted HR for death and MACE 2.45 and 1.74
Groepenhoff et al. (19)	CCTA CMR integrated	Calculate the incidence of macrovascular and microvascular disease in women and men, develop a decision-support tool	400 patients expected	ML	Actively recruiting participants
Dekker et al. (20)	LDACT during MPI	Investigate the association of automated CAC scores and MACE	747 patients	CNN	High CAC scores has HR of 2.19 in predicting MACE

*CT, computed tomography; FFR, fractional flow reserve; RNN, recurrent neural network; RF, random forest; MACE, major adverse cardiovascular event; CAG, coronary angiography; HR, hazard ratio; ACS, acute coronary syndrome; CNN, convolutional neural network; FSS, functional syntax score; OR, odds ratio; AUC, area under curve; CI, confidence interval; PCAT, pericoronary adipose tissue; CAD, coronary artery disease; ML; FRP, fat radiomic profile; CMR, cardiac magnetic resonance; SVM, support vector machine; LGE, late gadolinium enhancement; MVO, microvascular obstruction; MBF, myocardial blood flow; MPR, myocardial perfusion reserve; LDACT, low dose attenuation computed tomography; MPI, myocardial perfusion imaging; CAC, coronary artery calcium*.

**Table 3:** Applications of AI in invasive modalities.

**References**	**Modality**	**Purpose**	**Samples**	**Algorithm**	**Results**
Howard et al. (21)	CAG	Identify damping in arterial pressure waveform	5,709 beats	CNN	Sensitivity 100%, specifcity 99.8%, positive predictive value 98.1%, negative predictive value 99.5%
Moon et al. (22)	CAG	Recognize and localize stenosis	452 movie clips	CNN	Frame-wise AUC 0.971, frame-wise accuracy 0.934, clip-wise accuracy 0.965
Roguin et al. (23)	CAG	Estimate FFR	31 patients	ANN	Sensitivity 88%, specificity 93%, positive predictive value 94%, negative predictive value 87%
Yabushita et al. (24)	CAG	Detect stenosis	199 patients, 1,838 videos	CNN	Predictive accuracy: AUC 0.61
Zhao et al. (25)	CAG	Calculate FFR	137,126 images	CNN	Correlation between CFRauto and CFRmanual: *r* = 0.51
Du et al. (26)	CAG	Comprehensive analysis	20,612 angiograms	GAN CNN	F1-scores:stenosis, 0.829; total occlusion, 0.810; calcification, 0.802; thrombosis, 0.823; dissection, 0.854.
Lee et al. (27)	OCT	Developed an automated atherosclerotic plaque characterization method	6,556 images	CNN RF	Sensitivities/specificities: fibrolipidic plaques 84.8/97.8% fibrocalcific plaques 91.4/95.7%
Chu et al. (28)	OCT	Automatically characterize OCT plaques	509 pullbacks	CNN	Diagnostic accuracy: fibrous plaque 97.6%, lipid 90.5%, calcium 88.5%
Xu et al. (29)	OCT	Identify fibroatheroma with deep features	360 images	AlexNet, VGG-16, VGG-19, and GoogLeNet; SVM	Classification accuracy: Alexnet 0.7333, VGG-16 0.7611, VGG-19 0.7639, GoogLeNet 0.7333
Prabhu et al. (30)	OCT	Identify fibrolipidic and fibrocalcific A-lines in OCT images	6,556 images	SVM	Overall accuracy 81.58% sensitivity/specificity: other (81.43/89.59), fibrolipidic (94.48/87.32), fibrocalcific (74.82/95.28)
Shi et al. (31)	OCT	Boost the performance of recognizing vulnerable plaques	2,300 images	Fully CNN Deep CNN	Final score:0.8767
Liu et al. (32)	OCT	Improve the detection quality of vulnerable plaque	2,300 images	Deep CNN	Precision rate 88.84%, recall rate 95.02%, overlap rate 85.09%; detection quality score 88.46%
Lee et al. (33)	OCT	Characterize coronary calcified plaque in OCT images	8,231 images	CNN	Sensitivity 97.7%, specificity 87.7%, F1 score 0.922
Cha et al. (34)	OCT	Compare OCT-FFR with wire-based FFR	125 patients	RF	Sensitivity 100%, specifcity 92.9%, positive predictive value 87.5%, negative predictive value 100%, and accuracy 95.2%
Johnson et al. (35)	OCT	Use transcriptomic data to predict FCT change	69 patients	Elastic net K top scoring pair	Classification AUC = 0.969 and 0.972
Bae et al. (36)	IVUS	Develop ML models for predicting OCT-TCFA	517 patients	ANN SVM naïve Bayes	ANN: 81 ± 5% (AUC = 0.80 ± 0.08) SVM: 77 ± 4% (AUC = 0.74 ± 0.05) naïve Bayes: 78 ± 2% (AUC = 0.77 ± 0.04)
Jun et al. (37)	IVUS	Find the most accurate classifier to classify TCFA	12,325 images	FNN KNN RF CNN	AUC of: FNN:0.859, KNN:0.848, RF:0.844, CNN:0.911
Cho et al. (38)	IVUS	Develop IVUS-based algorithms for classifying attenuation and calcified plaques	113,746 frames	EfficientNet	Angle level dice similarity coefficients: calcification 0.79, attenuation 0.74 Frame level accuracy:attenuation 93%, calcification 96% Vessel level correlation with human measurment: attenuation *r* = 0.89, calcification *r* = 0.95
Wang et al. (39)	IVUS	1. Identify the most powerful predictor(s) for plaque vulnerability change 2. Test whether machine learning approaches could improve prediction accuracy	9 patients	SVM RF	Prediction accuracy: RF 91.47% SVM 90.78% MPVI the best single risk factor

*CAG, coronary angiography; ANN, artificial neural network; GAN, generative adversarial network; CFR, coronary flow reserve; OCT, optical coherence tomography; FCT, fibrous cap thickness; IVUS, intravascular ultrasound; TCFA, thin cap fibroatheroma; FNN, feed-forward neural network; KNN, K-nearest neighbor; MPVI, morphological plaque vulnerability index*.

## Applications OF AI in Non-invasive Imaging Modalities

### CT

#### CT-Derived FFR

CCTA has long been found to be a reliable method to give ACS an all-around evaluation. Cardiologists have used it to gain information, for example, relating to stenosis, calcification, plaque, lipid, and stent (40, 41). In recent years, a novel tool called computed tomography angiography-based fractional flow reserve (FFRCT) has emerged as a non-invasive alternative to traditional FFR obtained by pressure wire based on invasive CAG. Given that FFRCT involves a large amount of data collection and processing, artificial intelligence appears to have great potential for accomplishing such tasks.

Liu et al. acquired FFRCT in 243 symptomatic coronary artery disease (CAD) patients with deep learning (DL). In patients who had revascularization, major adverse cardiovascular event (MACE) rates in those with a DL-FFRCT value ≤ 0.8 (2.9%) were similar to those who had CAG-guided interventions (3.3%). If a DL-FFRCT value >0.8 was interpreted as positive, calling for intervention as high as 72% of CAG should not be done (5).

According to recent studies (6, 7), CT-FFR could not only assist in deciding the intervention but also predict prognosis, as a non-invasive alternative to traditional FFR. Driessen et al. found the Pearson's and Spearman's correlation coefficients between CT-FFR and wire-based FFR were 0.80 and 0.67, respectively (42) although the automatic method was different in various studies.

Qiao et al. designed a “Functional SYNTAX score” (FSSCTA) to forecast prognosis in patients with three-vessel CAD. FSSCTA is a combination of anatomical characteristics and functional characteristics produced by machine learning (ML)-based CT-FFR evaluation. The MACE predicting ability of FSSCTA was compared with that of SSCTA and SSICA (based on CAG). The predictive accuracy of FSSCTA for MACE proved to be better. Revascularization strategies guided by traditional SS and FSSCTA were also compared. With FSSCTA, 52 (22.9%) patients initially indicated for CABG guided by SSCTA would have been recommended to PCI (9).

With its non-invasive nature, CT-FFR significantly reduces costs by avoiding unnecessary CAG, as well as unnecessary anxiety and fear. Risk stratification tools should also consider updates by integrating with CT-FFR due to its easy accessibility and predictive power.

#### Markers Based on CT

Coronary artery calcium (CAC) has been shown to be powerful in predicting the extent and severity of CAD in symptomatic patients (43–45). Syntax score is used by interventionists and surgeons to assist in determining treating strategy as well as predicting outcome in patients who have had 3-vessel or left main CAD (46, 47). Studies have developed markers like CAC and Syntax score to assist in decision-making, prognosis predicting, and risk stratification, taking advantage of advances in artificial intelligence that have grown in recent decades.

A deep Convolutional Neural Network (CNN) is a powerful algorithm in image featuring. Zeleznik et al. confirmed that calcium on CCTA can be quantified automatically, moreover, the calcium score based on CNN can predict the outcome. CT readers localize the heart in cardiac CT, then segment the heart and the segment containing calcium, followed by the deep learning system automatically identifying and quantifying calcium. The AUC of the automatic method and manual approach was not different (8).

XGBoost is commonly applied in building predictive models, with the advantage of making weak classifiers into one single strong classifier. Featuring CT- based plaque qualitatively and quantitatively, Al' Aref et al. identified precursors of culprit lesion (CL) in ACS patients who had CAG. XGBoost algorithm was used to build a model predicting CLs. The predictive model performed well in discriminating CL precursors. The model also showed a specificity of up to 89.3% when tested in the non-ACS cohort (10). It may provide new insights into the target of secondary prevention of ACS.

Since XGBoost can identify culprit lesions, it can predict certain diseases theoretically, given the right data. CT radiomics have drawn attention from researchers who have then built models. Lin et al. successfully identified acute myocardial infarction (AMI) by building a machine learning model that combined a series of clinical factors and pericoronary adipose tissue attenuation with CT radiomic to identify AMI patients, achieving an AUC of 0.87 (11). With the help of powerful tools like XGBoost, radiomics can be of enormous use on multiple levels. We can expect that image biomarkers derived from CT radiomics will contribute to more precise diagnoses, risk stratification (29), and even clinical recommendations in the future.

Serum biomarkers also possess predicting capability, just like imaging biomarkers. XGboost was again proven to be capable of predicting cardiac events based on serum biomarkers integrating other data (12). We look forward to seeing what radiomics combining serum biomarkers can achieve.

#### Outcome Prediction

Atherosclerotic plaque features and stenosis can be evaluated qualitatively and quantitatively, providing comprehensive information to clinical practitioners, further enabling the prognostic prediction of ACS events (48–50).

A functional assessment like FFR and adverse cardiac events can both be predicted by CCTA features. Deciding on these features in a traditional way involves enormous statistical analysis. However, ML algorithms can finish the job, not only saving time and costs but also providing important information on potential intervening targets.

Yang et al. used Boruta and hierarchical clustering to identify the relevant features correlated with low FFR. They then assessed the ability to predict vessel-level adverse incidents in 5 years. In total, six features were identified as associated with low FFR. With the 6 relevant features increasing, the risk of vessel level adverse incidents in 5 years increased. Additionally, it is a better prognostic predictor than percent diameter stenosis and FFR (13). Additionally, random forest (RF) can also pick up features out of 1,000 radiomics which can strengthen the power of predicting MACE (14).

### Applications of AI in MR

Cardiac magnetic resonance (CMR) plays a pivotal part in comprehensively evaluating myocardial infarction (51). Cine and late gadolinium enhancement (LGE) sequences are most frequently referred to in this context. Functional evaluation is mainly performed by cine sequences because the movement can be captured by them (52). Detection of myocardial injury makes LGE sequence critical in diagnosing myocardial infarction (53).

#### Differentiating Diagnosis

In patients in whom AMI is complicated by chronic myocardial infarction (CMI) it is crucial to distinguish AMI and CMI for the sake of treatment and follow-up. However, ECG and coronary angiography provide limited information to pinpoint acute injury.

Larroza et al. used machine learning and extracted texture features in CMR images from 22 AMI patients and 22 CMI patients. They analyzed cine and LGE MRI separately to classify AMI and CMI. By evaluating the classification performance of three predictive models based on ML extracting texture features, the best performance was yielded by the polynomial SVM. It was demonstrated that feature analysis can be applied in differentiating AMI from CMI on both cine and LGE CMR (15). The imaging features that can separate the two groups were carefully selected by SVM classifier.

#### Predicting Prognosis

CMR is regarded to be a gold-standard non-invasive modality for assessing cardiac function quantitatively and characterizing myocardium after MI (54, 55).

One of the reasons artificial intelligence was developed is that it can replace human resources to some extent, on the premise that it can finish a human's job just as well, if not better. The CMR parameters of both ventricles can be analyzed both manually and computationally. Schuster et al. proved that automatic ventricle evaluation can predict MACE as well as manual evaluation. Volume parameters like left ventricular mass left and right ventricular ejection fraction and so on were automatically and manually analyzed. Parameters then entered regression models to predict MACE (16).

In a study conducted by Ma et al., 68 patients had CMR after PCI for AMI. The evaluation of the myocardial damage and prediction of left ventricular (LV) systolic contractility recovery were evaluated with radiomics signatures extracted by open-source software combining selected strongest features. Better diagnostic performance for microvascular obstruction (MVO) than T1 values alone was achieved by incorporating radiomics and T1 values. A greater predicting power for LV contractility recovery was also yielded by radiomics signature adding to T1 values compared to T1 values alone (17).

Derived from CMR perfusion images, myocardial stress-related metrics are used to predict MACE. During the process of deriving stress metrics, CNN was used to segment the contour of the ventricle and myocardium (18). Another example of artificial intelligence participating in predicting prognosis was exhibited.

### Application of AI in Other Non-invasive Modalities

Various modalities other than those above-mentioned examples have been used to prevent, diagnose, and treat ACS. Additionally, some methods integrate the above-mentioned modalities serving the same purpose as the tools of machine learning.

Integration of CCTA, stress CMR perfusion imaging, and electronic medical record data is proposed for building decision-making assisting systems (19). Machine learning is destined to play a role in these systems, although it is uncertain which algorithm will be used.

Dekker et al. used deep learning on low-dose attenuation correction CT (LDACT) images from 747 patients with chest pain gathered during 82 Rubidium PET/CT in one single assessment, to get CAC scores. High CAC scores (>400) showed the higher predictive value of events. Both high CAC scores and ischemia were found to be independent predictors of MACE (20). This demonstrates that deep learning methods can also be applied to imaging systems derived from PET/CT.

## Application of AI in Invasive Modalities

### CAG

#### Arterial Waveform Analysis

Howard et al. implemented a 1-dimensional convolutional neural network to automatically analyze arterial pressure waveforms. With the algorithm, real-time identification of damping can be realized to guarantee the safety of intervention for ACS patients. The classification network achieved excellent accuracy, specificity, sensitivity, positive, and negative predicting values (21). This indicates that, given the right circumstances, artificial intelligence can serve us in many ways.

#### Stenosis Recognizing

If we analyze CAG images with neural networks like ANN or CNN, in theory, stenosis or thrombus or calcification will be identified given sufficient labeling. Stenosis, as the most significant information extracted by interventionists, has naturally become the primary subject.

Moon et al. designed a three-step algorithm to recognize stenosis in coronary angiography automatically. The model was trained with 452 series of right coronary angiography. In internal and external validation sets, both frame-wise and series-wise satisfactory accuracy were achieved (22).

Yabushita et al. attempted to detect clinically significant stenosis in coronary angiography movies with a model. One hundred and ninety-nine patients with 1,838 movies were enrolled to produce the multi-layer 3D CNN model. A c-statistic value of 0.61 was achieved in the test set as well as the validation cohort in the training set (24).

#### CAG-FFR

ANN classifies lesions, as stated above. What surprised us is that software based on ANN can furthermore compute FFR instantaneously without any additional movement, while the respective vessel is being viewed (23). CNN was applied for the same purpose and achieved acceptable correlation (25).

#### Comprehensive Analysis

Since deep neural network (DNN) is widely used in CAG image processing, is it applicable for finishing comprehensive analysis, from segmentation to stenosis measurement, from calcification to dissection. Du et al. implemented two different DNNs to accomplish such tasks, which only took seconds. The labeling of coronary artery segments and lesion types is a key factor in training the network (26). We can expect to have a full analysis of CAG almost instantaneously for interventionists to make decisions in the near future.

### OCT

#### Plaque Analysis

Owing to high spatial resolution (15 μm), OCT has an inherent advantage in morphological analysis of plaques (56, 57). Studies focusing on plaque characteristics or classification have grown in recent years. Accurately and efficiently identifying atherosclerotic plaques, in particular, vulnerable plaques which are often an alarming sign of successive cardiac events, which are of great significance in managing ACS (58). Additionally, with AI's prosperity, OCT combining AI are the proposed solutions to a series of clinical challenges.

CNN has repeatedly demonstrated its classifying ability in other imaging modalities and has also been proven in OCT. Furthermore, RF was combined with CNN, also serving as a classifier. For starters, RF is particularly efficient in a large data set. Secondly, RF has a relatively low risk of overfitting. Thirdly, RF is good at deciding the significance of features that matter in classification. Lastly, RF is robust in noisy data and OCT data is “noisy.” As a result, Lee et al. mixed DL and manual lumen morphological characteristics to automatically feature atherosclerotic plaques. High sensitivities and specificities for fibrolipidic and fibrocalcific plaques were achieved after sequential pre-processing, training, testing, and post-processing. The hybrid approach performed better than the previous automatic or manual method alone. The training also depends on accurate labeling (27).

CNN was then widely tested in OCT modality to classify different plaques (28, 59), including vulnerable plaques (31, 32). SVM was also tested in the OCT modality to classify fibrolipidic and fibrocalcific plaques (30). If calcified plaque is the focus of classification, CNN could also accomplish the task, furthermore, to pursue excellence, other DL techniques can be integrated (33).

#### OCT-Based FFR

FFR is considered a highly specific tool for diagnosing myocardial ischemia in borderline angiographic stenosis (60, 61). However, it provides no information on the morphology of lesion characteristics like OCT. Researchers have sought to combine FFR with OCT via the application of artificial intelligence to acquire both functional and morphological information at the same time. Cha et al. obtained OCT data from 125 patients with typical angina and left anterior descending artery lesions of borderline stenosis (luminal diameter <70%), as well as their FFR data. Random forest extracted the six most important features to predict FFR. The OCT-based ML-FFR correlated well with the wire-based FFR (34). As introduced in the previous sections discussing CT-FFR, most FFR calculations are merely based on images but prediction models integrating both imaging and clinical data broaden our vision.

#### Predicting FCT Change

Fibrous cap thickness (FCT) precisely measured by OCT (56) is of utmost the importance in plaque rupture (62, 63). Statin is believed to make FCT grow so that acute coronary events are less likely to occur (64, 65). Nevertheless, statin is not effective in everyone by showing increased FCT (66). Hence a tool to predict FCT change in patients taking statin will undoubtedly optimize medical therapy in CAD patients to reduce incidents of ACS. ML models predict FCT changes measured by OCT via analyzing gene expression data (35). Once models like this are integrated, precision medicine can potentially be practiced.

### IVUS

#### Plaque Analysis

According to previous studies, large lipid core and thin fibrous cap can independently predict cardiac events including ACS (58). Intravascular ultrasound (IVUS) is widely used in evaluating lesions and plaque. However, conventional frame-by-frame analysis is not efficient. Various artificially intelligent algorithms have been sought to assist in analyzing plaques.

High risk plaques are undoubtedly the first-choice target for classifying models in IVUS modality. A computational method called EfficientNet was introduced to identify “attenuated plaque, calcified plaque, and plaque without attenuation or calcification” (38). This novel approach has potential and may be of assistance in “high risk” plaque recognition.

Thin-cap fibroatheroma (TCFA) is defined as “a lipid-rich plaque underlying a thin-fibrous cap whose thickness is <65 μm” (67). The existence of TCFA independently predicts adverse cardiac events, especially ACS, as concluded by a few studies looking into the progress of non-culprit lesions. However, the relatively poor resolution of IVUS makes it impossible to identify TCFA. ML have appeared in predicting and classifying TCFA for its capability in finding patterns in a huge dataset and precise prediction with processed data.

Bae et al. collected IVUS and OCT images in patients with stable and unstable angina, respectively. They then separated them into the training and testing samples. Each of the IVUS-OCT co-registered frames was labeled as with TCFA and without TCFA. ANN, SVM, and naïve Bayes were used to predict OCT-derived TCFA, all of which showed accuracies of around 80% (36). Other forms of neural networks were also proven to possess similar capability (37).

#### Plaque Vulnerability Prediction

A genuine clinical challenge arises in predicting upcoming plaque rupture and related critical events like myocardial infarction. To solve this, some effort has been made to take advantage of artificial intelligence for its strength in image feature extraction, a huge quantity of data processing, complex pattern finding, and biomechanics for its advantage in studying the fluid environment in which vulnerable plaques reside in the perspective of fluid mechanics.

A “morphological plaque vulnerability index (MPVI)” has been proposed to evaluate plaque vulnerability using morphological features was obtained from *in vivo* IVUS images. Wang et al. acquired IVUS data from nine patients to reconstruct “fluid-structure interaction (FSI)” models in which hydrodynamic metrics were obtained. In total, 10 baseline risk factors were used by three models to forecast “MPVI change (ΔMPVI = MPVI_follow−up_ – MPVI_baseline_).” Model of RF performed best and MPVI was weighed most in the predictors (39).

## Pitfalls and Limitations

All kinds of novel methods/algorithms/models appear to be promising but there is still much work to be done before they can be translated for clinical use. No matter how well the newly developed models perform, strict external validation with cohorts from different centers other than the centers where that model was built is mandatory. Before proving satisfactory generalizability, clinical deployment is, at present, out of the question.

Overfitting is a common trap in a complex algorithm, although various techniques can be applied to avoid it. A model with good performance may yield misleading results when applying new data, leading to serious sequelae because of overfitting. Therefore, techniques like K-fold cross validation should be considered to reduce errors in prediction or pattern finding.

One of the major limitations of building an ML model is the quality of data. The incorrect selection of data and inaccurate measurements may produce flawed results that could be misleading. The same problem also applies to data that have too much noise.

Involving big data processing and a huge amount of calculations, conducting AI research undoubtedly involves demands both in terms of software and hardware. Further advances in AI study are anticipated but require a lot of investment.

To date, no guidelines or expert consensus has been issued. Standardization of AI research is urgently required to guarantee the quality of AI research.

## Challenges and Directions

Essentially AI is a science based on data. Generally speaking, more data means better AI research products. Although AI researches are prospering, the scarcity of data remains a challenge. One important reason for this is that clinical data often are stored in different systems. For example, images are in Picture Archiving and Communication Systems (PACS), electronic health records are stored in Hospital Information Systems (HIS), and electrocardiograms (ECG) are in paper format. Collecting integrated clinical data is therefore time consuming and demanding of human resources. A revolutionary data storing system is required in order to tackle this obstacle.

Legislation focusing on clinical AI products is still in development in most countries. Due to the complexity of AI in terms of legal and ethical issues, the process of legislation is expected to be long-term and difficult, given that there is no precedent in human history. There will likely be polarizing debates about whether the developer, the user, or the AI itself are accountable when the AI model produces negative results in the real world.

There have already been products integrating the collection of health-related information, such as smart wearable devices and hand-held diagnostic tools. Mobile devices possess an inherent advantage for obtaining clinical data. In the future, it is likely to be a popular direction with the potential to develop more accurate disease phenotyping and more personalized therapies.

It is noteworthy that ACS often requires timely management and AI products involving treatment schedules should take processing time into account. Similar to the example algorithms mentioned above, it is best to be able to display results simultaneously or within seconds along with the CAG or PCI.

Attempts have been made to integrate different imaging modalities to evaluate ACS comprehensively with efficiency in terms of time and cost, such as IVUS and OCT in fusion. Although large clinical trials are lacking, they may also be a prospective direction.

## Conclusion

Many gaps are to be bridged in cardiovascular disease, ACS in particular, from the mechanism of disease to precise diagnosis and personalized optimal therapeutic strategy. AI has shown its potential in making accurate diagnoses, evaluating functions precisely, predicting risk and outcome, assisting in making treatment decisions, and monitoring disease progression, etc. based on its inherent advantages compared to human power. However, AI also has limitations to be addressed before being widely deployed clinically. Strenuous effort should be made to tackle overfitting, lack of generalizability, limited interpretability, robustness, and so on. Meanwhile, standardization of conducting AI research is an urgent matter. The application of AI to cardiovascular medicine in the future will provide supplemental options for clinicians and benefits to patients.

## Author Contributions

M-hL constructed the writing of the article. CZ made major revision on the structure and mostly made the Central figure. SW made major revision on the writing. All work were under HJ and BY's guidance. All authors contributed to the article and approved the submitted version.

## Funding

This work was supported by the National Key R&D Program of China (grant No. 2016YFC1301104 to BY) and the National Natural Science Foundation of China (grants: 81722025, 82061130223, and 81827806).

## Conflict of Interest

The authors declare that the research was conducted in the absence of any commercial or financial relationships that could be construed as a potential conflict of interest.

## Publisher's Note

All claims expressed in this article are solely those of the authors and do not necessarily represent those of their affiliated organizations, or those of the publisher, the editors and the reviewers. Any product that may be evaluated in this article, or claim that may be made by its manufacturer, is not guaranteed or endorsed by the publisher.
